# The association between the lifestyle risk score and metabolically healthy and unhealthy obesity phenotype in Iranian women with overweight and obesity: a cross-sectional study

**DOI:** 10.3389/fpubh.2025.1490937

**Published:** 2025-02-14

**Authors:** Sara Ebrahimi, Farideh Shiraseb, Maryam Ladaninezhad, Azimeh Izadi, Negin Navaei, Khadijeh Mirzaei

**Affiliations:** ^1^Institute for Physical Activity and Nutrition (IPAN), School of Exercise and Nutrition Sciences, Deakin University, Geelong, VIC, Australia; ^2^Department of Community Nutrition, School of Nutritional Sciences and Dietetics, Tehran University of Medical Sciences (TUMS), Tehran, Iran; ^3^Medical Department, Orchid Pharmed Company, Tehran, Iran; ^4^Department of Nutrition, College of Graduate and Undergraduate Studies, Life University, Marietta, GA, United States

**Keywords:** lifestyle risk score, obesity phenotypes, metabolically healthy obesity, metabolically unhealthy obesity, healthy lifestyle

## Abstract

**Background:**

The evidence shows that all women with obesity do not develop metabolic complications thus, they may be metabolically healthy. The lifestyle factors in combination may influence obesity phenotypes including metabolically healthy and unhealthy obesity. While previous studies examined associations between single lifestyle factors and obesity phenotype, no previous study has examined associations between lifestyle risk score (LRS) and obesity phenotypes. This study for the first time created the LRS which is a combination of lifestyle factors and investigated the LRS in relation to various obesity phenotypes among women with overweight and obesity.

**Methods:**

This cross-sectional study analyzed 278 women referred to health centers of the Tehran University of Medical Sciences. A multistage sampling method was used to recruit the participants. The LRS was created based on diet, physical activity (PA), sleep, obesity, and sociodemographic characteristics. A binary logistic regression analysis was used to evaluate the association between obesity phenotypes and LRS.

**Results:**

Women with higher LRS had higher body mass index (BMI) and high-sensitivity C-reactive protein (hs-CRP) while had lower high-density lipoprotein cholesterol (HDL-C), PA, education levels, sleep quality, vegetables, grains and legumes intake. Furthermore, women with higher LRS were more likely to experience metabolically unhealthy obesity (MUO).

**Conclusion:**

This study found significant associations between higher LRS and an increased likelihood of MUO. Further prospective studies are needed to advance our understanding of the relationship between lifestyle and obesity.

## Introduction

The global prevalence of overweight and obesity has risen steadily since 1975, affecting all regions (1). In particular, the Eastern Mediterranean Region (EMR) has undergone a significant nutrition transition in recent decades. Obesity stands out as a major health concern in EMR countries, including Iran (2). A systematic review and meta-analysis 2021, reported that the prevalence of overweight and obesity in Iranian adults was 35 and 21%, respectively (3). Notably, obesity rates are higher among Iranian women compared to men (4). Obesity is associated with metabolic disorders and non-communicable diseases (NCDs) such as diabetes, cardiovascular diseases, non-alcoholic fatty liver disease, and cancer (5). NCDs prevalence is increasing in the EMR countries, with NCDs being the major cause of 82% of deaths in Iran in 2020 (6). However, not all people with obesity develop metabolic complications (7). Some people with obesity are not experiencing dyslipidemia, hyperglycemia, and hypertension (8). This obesity phenotype is called metabolically healthy obesity (MHO). On the other hand, the metabolically unhealthy obesity (MUO) phenotype is the state of having at least two or more metabolic disorders (9). Based on the Karelis criteria, triglyceride (TG), low-density lipoprotein cholesterol (LDL-C), high-density lipoprotein cholesterol (HDL-C), hs-CRP, and homeostatic model assessment for insulin resistance (HOMA-IR) are involved in determining MHO (10, 11).

There is limited evidence on the determinant factors of MHO and MUO. The evidence shows that higher compliance with a healthier diet and higher physical activity (PA) levels are associated with a healthier metabolic state (12, 13). Furthermore, shorter sleepers are at a higher risk of developing metabolic complications (14).

The existing evidence shows that lifestyle-related factor including adiposity is associated with a higher risk of chronic diseases (15). Central body fat distribution and impaired adipose tissue function have been shown to be the predictors of obesity-related metabolic abnormalities (16). Also, a study reported that adults with lower levels of education and income are more likely to experience MUO (17).

Obesity is related to various lifestyle factors such as diet, PA, and sleep. As a result, several factors may simultaneously affect obesity. Lifestyle factors are often interconnected, with individuals typically adopting related lifestyle patterns (18, 19). As a result, lifestyle factors should be examined together to more effectively assess their impact on health (18, 19). *A priori* approach provides Information on a variety of lifestyle factors that can be described by a single score. This score could be used for assessing associations between healthy and unhealthy lifestyles and health outcomes (19, 20). While previous studies have explored the relationship between individual lifestyle factors and MHO and MUO, understanding how a combination of various lifestyle factors collectively influences obesity phenotypes remains limited. Holistic approaches such as *a priori* and posteriori methodologies offer a comprehensive perspective by examining lifestyle factors concurrently (21). Despite the importance of exploring the combined impact of different factors on MHO and MUO, there is a paucity of evidence in this area. A study on Lebanese adults with overweight and obesity in 2020 applied the factor analysis approach for the first time and assessed the association between lifestyle patterns and MHO and MUO and found a positive link between a healthy lifestyle pattern and MHO (22). Nevertheless, no prior study has employed an *a priori* approach to investigate the correlations between lifestyle scores and MHO and MUO. Consequently, this study is designed to explore the associations between lifestyle risk scores (LRS) and various obesity phenotypes among Iranian women with overweight and obesity. While Middle Eastern populations including Iranians have a particular type of obesity which is characterized by abdominal fat accumulation and greater waist circumference, the evidence on the lifestyle factors in relation to obesity phenotype is scant in this population. As a result, it is of significance to advance knowledge about this region (23).

## Methods

### Study design and population

This cross-sectional study was conducted on 278 Iranian women with overweight and obesity who were referred to health centers of the Tehran University of Medical Sciences (TUMS). A multistage random sampling method was used to recruit participants from the health centers in Tehran which were affiliated with TUMS. The inclusion criteria included adult women with a BMI of ≥25 kg/m^2^. Individuals with a history of cardiovascular diseases, stroke, kidney disorders, liver diseases, thyroid disease, inflammatory illnesses, cancer, pregnancy, lactation, and menopause were excluded. Menopause could significantly change body composition, metabolic and hormonal changes (24, 25). Furthermore, based on the previous studies, under and over reporters of energy intakes below 800 or exceeding 4,200 kcal/day along with those with ongoing weight loss programs or taking weight loss supplements, were excluded (26–28). Women who did not respond to more than 70 questions on the food frequency questionnaire (FFQ) were excluded from participating in this study (29). A trained nutritionist conducted all the face to face interviews on one visit. This study was approved by the Ethical Committee of the Tehran University of Medical Sciences (ethics number: IR.TUMS.VCR.REC.1398.142) (30, 31). All methods were performed in accordance with the relevant guidelines and regulations. This article is prepared based on the Strengthening the Reporting of Observational Studies in Epidemiology—Nutritional Epidemiology (STROBE-Nut) reporting checklist (32).

### Sociodemographic characteristics

A trained nutritionist collected the data on sociodemographic characteristics including age, education level, marital status, occupation, and income, using a questionnaire in an interview. Participants were grouped based on their education levels and employment status: high education level (bachelor degree and higher), low education level (diploma and lower), employed and non-employed. Participants were categorized into high-income (above the poverty line) and low-income (below the poverty line) based on the poverty line in Iran which was determined as 11 million and 500 thousand rials for each person in 2018. The variables including occupation, education and income, were used to measure the socioeconomic status (SES) (33). Based on the SES median score, participants were categorized into two groups: high SES (≥2) and low SES (<2). The Pittsburgh Sleep Quality Index (PSQI) was used to assess participants’ sleep quality including subjective sleep quality, sleep latency, sleep duration, habitual sleep efficiency, sleep disturbances, use of sleep medications, and daytime dysfunction. The PSQI score ranges between 0 and 21, and a total score over five indicates poor sleep quality (34). The International Physical Activity Questionnaire (IPAQ) was used to collect participants’ PA (35). PA >20 the metabolic equivalent of task (MET-h/week) and PA ≤ 20 MET-h/week, were considered high and low levels of PA, respectively.

### Anthropometric indices

A Seca digital scale (Germany) was used to measure participants’ body weight while the height was measured using a Seca 206 stadiometer (Germany) with a precision of 0.2 cm. Waist circumference (WC) and hip circumference (HC) were measured to the nearest 0.2 cm. The waist-to-height ratio (WHtR) was calculated as WC (cm) divided by height (cm). Given that WHtR is an indicator of early health risk linked to central obesity, two obesity categories were created; WHtR≤0.5 cm (non-obesity) and WHtR>0.5 (obesity), respectively (36, 37). Body composition was measured using a bioelectrical impedance analyzer (BIA; Inbody 770 Co., Seoul, Korea). Fat mass was measured using Dual Energy X-ray Absorptiometry (DXA) and bioelectrical Impedance Analysis.

### Dietary intake assessment

The frequency of each food consumed over the past year was collected using a 147-item semi-quantitative FFQ by a trained nutritionist. Participants reported the frequency of consumption for a given serving of each food item over the last year on a daily, weekly, monthly, or yearly basis. Portion size for the consumed food was converted to grams per day using household measurements (38). The NUTRITIONIST-IV (version 7.0; N Squared Computing, Salem, OR, United States) was used to measure the nutrients and energy intake. Considering the strong link between obesity and cardiovascular diseases, the American Heart Association (AHA) diet score was calculated for each participant (39, 40). The AHA components include fruits and vegetables, fish and shellfish, sodium, sugar-sweetened beverages, whole grains, nuts, seeds and legumes, processed meats, and saturated fats. Scores for each component ranged from 0 to 10, with a total score ranging from 0 to 80. Participants were categorized into two groups: those with lower adherence to dietary recommendations (< 40) and those with higher adherence to dietary recommendations (≥40) (41, 42) (Supplementary Table 1).

### Biochemical parameters

The blood test was taken after 10–12 h of fasting in the nutrition laboratory of the Tehran University of Medical Sciences. Fasting blood glucose (FBS), triglycerides (TG), and total cholesterol (TC) were measured using the glucose oxidase-phenol 4-amino antipyrine peroxidase (GOD-PAP) and glycerol-3-phosphate oxidase–phenol 4-amino antipyrine peroxidase (GPO-PAP) methods. Low-density lipoprotein (LDL) and high-density lipoprotein (HDL) cholesterol levels were determined through direct enzymatic clearance. The evaluation was based on the ratio of triglycerides (TG) to high-density lipoprotein cholesterol (HDL-C). Aspartate transaminase (AST) and Alanine transaminase (ALT) levels were measured based on the International Federation of Clinical Chemistry and Laboratory procedures. An immunoturbidimetric test with the Pars Azmoon kit (Pars Azmoon Inc. Tehran) was used to measure hs-CRP levels. Fasting blood sugar (FBS) was evaluated using the glucose Oxidase Phenol 4-Aminoantipyrine Peroxidase method. Insulin resistance (IR) was assessed based on the Homeostatic Model Assessment-Insulin Resistance (HOMA-IR) formula: fasting serum insulin (mIU/L) * FBS (mmol/L) / 22.5. The insulin levels were measured using a radio-immune assay method (28).

### LRS assessment

Based on the previous studies, lifestyle-related factors relevant to Iranians were selected (23, 43). The variables including sleep quality, SES, WHtR, dietary behavior and PA were used to create the LRS based on the previous studies (23, 43). Participants were scored 1 if the AHA diet score was <40, PA ≤ 20 MET-h/week, PSQI>5, WHtR>0.5, SES < 2, otherwise, there were scored 0.Participants were categorized as the high LRS group if the total LRS was higher than the median (score > 2). However, participants were classified as the low LRS group if the total LRS was lower than the median (score ≤ 2) (Supplementary Table 2).

### Metabolic health and its components

The metabolic healthy and unhealthy obesity were described based on the Karelis criteria which were used in the previous Iranian studies (28, 44, 45). The Karelis criteria have been utilized as they incorporate both insulin resistance and inflammation as practical indicators for assessing health in individuals with obesity (46). The participants were considered metabolically healthy subjects if they had four or more of the following components: triglycerides ≤1.7 mmol/L or use of lipid-lowering drugs, HDL ≥ 1.3 mmol/L, LDL ≤2.6 mmol/L, HOMA ≤2.7, and hs-CRP ≤3.0 mg/L. Otherwise, they were classified as metabolically unhealthy subjects (10, 11).

## Data analysis

The sample size was measured using the formula below: With *α* = 0.05, *β* = 0.95, and r = 0.25, n = [(Z 1 − α + Z 1 − β) × √1 − r2]/r 2 + 2). SPSS software version 26 was used to perform the data analysis. The Kolmogorov–Smirnov test was utilized to confirm the normal distribution of dependent variables. Continuous variables were presented as mean and standard deviation while categorical variables were presented as the number and percentage. The continuous variables between the two groups were compared using a one-way analysis of variance (ANOVA), while categorical variables were compared using the chi-square test. Analysis of covariance (ANCOVA) was used to adjust the analysis for potential confounders including age, energy intake, and BMI. A binary logistic regression analysis was used to evaluate the association between obesity phenotype categories (binary dependent variable) and LRS (continuous independent variable). The analysis was adjusted for age and energy intake. The 0.05 level of significance was considered as minimal statistical significance. A *p*-value <0.05 was considered statistically significant.

## Results

### The characteristics of participants

The mean age of participants was 36.2 years (± 8.4), with an average weight of 79.4 kg (± 10.8), a BMI of 30.5 kg/m^2^ (± 3.6), and a WC of 94.9 cm (± 15.6). The majority of participants were married (72%), had supplementation intake (58%), had moderate SES (46%), had high LRS (65%), and were MUO (73%).

### The characteristics of participants over the median of LRS

The association between the characteristics of participants over the median of LRS is presented in Table 1. After adjusting for age, energy intake, BMI, women with higher LRS had lower PA (*p* = 0.035), lower HDL (*p* = 0.041) and higher hs-CRP (*p* = 0.040) than women with lower LRS. The majority of women with higher LRS had lower education levels (*p* < 0.001) and poor sleep quality (*p* < 0.001).

**Table 1:** Characteristics of participants over the median of LRS (*n* = 278).

Variables	LRS		*p*-value	*p*-value*
	Low risk (*n* = 106) Median ≤ 2	High risk (*n* = 172) Median > 2		
	Mean ± SD			
Continuous variables, mean ± SD				
Age (year)	34.6 ± 8.1	36.4 ± 8.9	0.161	0.183
Weight (Kg)	77.8 ± 12.8	79.7 ± 8.9	0.260	0.369
Height (cm)	161.8 ± 5.2	160.7 ± 5.9	0.209	0.345
PA (MET min/ week)	1274.9 ± 1242.3	833.3 ± 1044.4	**0.018**	**0.035**
BMI (Kg/m^2^)	29.6 ± 4.2	30.8 ± 2.9	**0.021**	0.064
WC (cm)	92.6 ± 22.3	95.7 ± 8.5	0.293	0.298
Body fat mass (Kg)	31.4 ± 8.4	33.3 ± 6.1	0.082	0.220
FBS (mg/l)	85.9 ± 8.5	87.4 ± 9.8	0.333	0.499
TG (mg/l)	113.9 ± 61.8	124.3 ± 58.4	0.265	0.524
Chol (mg/l)	177.7 ± 32.0	180.7 ± 32.6	0.545	0.839
HDL (mg/l)	49.3 ± 10.5	46.3 ± 9.5	0.055	**0.041**
LDL (mg/l)	93.7 ± 21.3	99.4 ± 21.9	0.093	0.263
hs-CRP (mg/l)	3.2 ± 3.7	5.2 ± 5.3	**0.008**	**0.040**
Categorical variables, n (%)				
Supplementation intake, N (%)				
Yes %	35 (36.1)	62 (63.9)	0.872	0.710
No %	25 (37.3)	42 (62.7)		
Educational status, N (%)				
Diploma and under Diploma	16 (18.0)	73 (82.0)	**< 0.001**	**< 0.001**
Bachelor and higher	50 (56.8)	38 (43.2)		
Marital status, N (%)				
Single	19 (37.3)	32 (62.7)	0.984	0.933
Married	46 (37.1)	78 (62.9)		
Economic status, N (%)				
Poor	17 (36.2)	30 (63.8)	0.429	0.546
Moderate	27 (34.6)	51 (65.4)		
Good	22 (45.8)	26 (54.2)		
Sleep quality, N (%)			**<0.001**	**<0.001**
Poor	22 (20.4)	86 (79.6%)		
Good	42 (50%)	42 (50%)		

BMI, body mass index; chol, Cholesterol; FBS, fasting blood sugar; HDL, High-density lipoprotein cholesterol; HOMA-IR index: Homeostatic Model Assessment of Insulin Resistance index; LDL, Low-density lipoprotein cholesterol; LRS, lifestyle risk score; MET, metabolic equivalent of task; PA, physical activity; WC, waist circumstance; WHR: waist to hip ratio, TG, Triglycerides. Values are represented as means and SD and number (%) for continuous and categorical variables, respectively. *p-value obtained from ANCOVA. The analysis was adjusted for age, energy intake, BMI. *p* < 0.05 were considered as significant. BMI was considered the collinear variable for body composition, and anthropometric measurements. The bold values are statistically significant *p* < 0.05.

### Dietary intakes over the median of LRS

Participants’ dietary intake over the median of LRS is presented in Table 2. Women with higher LRS had a lower intake of vegetables (*p* = 0.038), whole grains (*p* = 0.007), and legumes (*p* = 0.006) than women with lower LRS.

**Table 2:** Dietary intake over the median of LRS (*n* = 278).

Variables	LRS		*p*-value	*p*-value*
	Low risk (*n* = 106) Median ≤ 2	High risk (*n* = 172) Median > 2		
	Mean ± SD			
Macronutrients and energy				
Energy intake (kcal/d)	2653.2 ± 781.3	2584.1 ± 741.4	0.558	–
Cho (% TEI)	56.8 ± 6.2	56.9 ± 6.8	0.859	0.945
Fat (% TEI)	31.9 ± 6.1	32.2 ± 6.2	0.750	0.674
Protein (% TEI)	14.3 ± 2.4	13.7 ± 2.3	0.245	0.249
SFA (mg/d)	27.8 ± 10.4	28.9 ± 12.4	0.560	0.248
PUFA (mg/d)	19.6 ± 9.3	20.4 ± 7.9	0.547	0.422
MUFA (mg/d)	30.6 ± 11.5	31.4 ± 11.0	0.859	0.211
Trans fat (g/d)	0.0 ± 0.0	0.0 ± 0.0	0.325	0.355
Total fiber (g/d)	45.2 ± 18.8	44.5 ± 18.9	0.812	0.686
Linoleic acid (g/d)	16.7 ± 8.9	17.8 ± 7.6	0.390	0.324
Linolenic acid (g/d)	1.3 ± 0.7	1.2 ± 0.6	0.324	0.304
EPA (g/d)	0.0 ± 0.0	0.0 ± 0.0	0.435	0.621
DHA (g/d)	0.1 ± 0.1	0.1 ± 0.1	0.476	0.666
Micronutrients				
Vit A (mg/d)	813.9 ± 431.6	751.4 ± 380.4	0.316	0.395
Vit C (mg/d)	188.3 ± 116.1	197.2 ± 148.5	0.676	0.436
Vit E (mg/l)	15.9 ± 8.0	17.9 ± 9.0	0.123	0.111
Ca (mg/d)	1184.9 ± 472.5	1155.6 ± 406.2	0.663	0.976
Iron (mg/d)	19.6 ± 6.5	18.3 ± 5.8	0.171	0.185
Thiamin (mg/d)	2.1 ± 0.7	2.1 ± 0.6	0.602	0.942
Niacin (mg/d)	26.0 ± 9.4	24.5 ± 8.9	0.300	0.603
Riboflavin (mg/d)	2.2 ± 0.8	2.2 ± 0.9	0.977	0.465
Vit B5 (mg/d)	6.8 ± 2.4	6.4 ± 2.8	0.308	0.470
Vit B6 (mg/d)	2.3 ± 0.8	2.1 ± 0.7	0.087	0.068
Biotin (mg/d)	40.3 ± 2.9	4.3 ± 1.9	0.235	0.460
Folate (mcg/d)	629.7 ± 193.7	589.3 ± 169.3	0.147	0.240
Vit B12 (mcg/d)	4.6 ± 2.9	4.3 ± 1.9	0.371	0.433
Zinc (mg/d)	13.7 ± 4.4	12.6 ± 4.3	0.105	0.060
Copper (mg/d)	2.1 ± 0.7	1.9 ± 0.8	0.224	0.353
Selenium (mg/d)	122.4 ± 42.3	117.6 ± 39.9	0.459	0.821
Chromium (mg/d)	0.1 ± 0.1	0.1 ± 0.1	0.300	0.477
Caffeine (mg/d)	127.7 ± 83.9	147.3 ± 105.9	0.201	0.142
Food groups				
Fruits (g/d)	545.9 ± 341.3	502.4 ± 329.8	0.669	0.908
Vegetables (g/d)	501.6 ± 289.5	403.5 ± 234.5	**0.031**	**0.038**
Whole grains (g/d)	9.8 ± 11.9	5.3 ± 7.6	**0.005**	**0.007**
Nuts (g/d)	17.0 ± 16.9	12.7 ± 14.7	0.111	0.138
Legumes (g/d)	67.3 ± 51.6	45.9 ± 33.6	**0.004**	**0.006**
Eggs	23.6 ± 13.2	19.8 ± 14.3	0.033	0.041
Refined grains (g/d)	443.4 ± 245.4	429.3 ± 192.6	0.243	0.309
Dairy (g/d)	418.3 ± 273.9	382.4 ± 226.7	0.667	0.820
Red meat (g/d)	443.4 ± 245.4	429.3 ± 192.6	0.080	0.099
White meat (g/d)	51.7 ± 45.2	45.4 ± 49.9	0.233	0.256
Sea foods (g/d)	12.5 ± 13.5	10.6 ± 11.4	0.779	0.720

Cho, Carbohydrate; DHA, Docosahexaenoic acid; EPA, eicosapentaenoic acid; LRS, lifestyle risk score; MUFA, monounsaturated fatty acid; SFA, saturated fatty acid; Pro, protein; PUFA, polyunsaturated fatty acid; TEI, Total energy index. Values are represented as means (SD). *p*-value obtained from ANVOVA test. The analysis was adjusted for macronutrients and micronutrients and energy intake. *p* < 0.05 were considered as significant. The bold values are statistically significant *p* < 0.05.

### The association between LRS and obesity phenotype

The association between LRS and obesity phenotype is found in Table 3. Women with higher LRS were more likely to be MUO (OR = 2.5, 95% CI = 1.3, 4.9, *p* = 0.01). After controlling for age and energy intake, there was a significant positive association between higher LRS and MUO (OR = 2.4, 95% CI = 1.2, 4.7, *p* = 0.01).

**Table 3:** Associations between obesity phenotypes and LRS (*n* = 278).

Obesity phenotype MUO	High LRS					
	Crude model			Model 1		
	OR	95% CI	*p* value	OR	95% CI	*p* value
	2.5	1.3, 4.9	**0.01**	2.4	1.2, 4.7	**0.01**

CI, confidence interval; LRS, lifestyle risk score; MUO, metabolic unhealthy obesity. *p*-value was obtained using binary logistic regression. The odds ratio (OR) has been reported. Model 1: The analysis was adjusted for age, and energy intake. Metabolic healthy phenotype was considered a reference group. Low LRS was considered a reference group. *p* < 0.05 were considered significant. The bold values are statistically significant *p* < 0.05.

## Discussion

To the best of our knowledge, this study, for the first time, applied *a priori* approach to examine the LRS in relation to obesity phenotype. For developing the LRS, dietary intake, PA, sleep quality, WHtR, and SES were involved. The primary finding indicates that women with higher LRS are more likely to experience MUO.

This study demonstrated that women with higher LRS were at a greater risk of developing MUO. Our findings underscore the impact of lifestyle factors on obesity, particularly the role of PA. Women with higher LRS, indicative of unhealthier lifestyles, demonstrated lower levels of PA. The evidence consistently supports the positive influence of PA on metabolic health, with benefits including improved lipid profiles, insulin sensitivity, glucose uptake, and reduced blood pressure and inflammation (13, 47, 48). Furthermore, PA levels are found to be inversely associated with CRP levels (49). While no previous study assessed the LRS in relation to obesity phenotype, only one study employed an empirical approach and examined a combination of lifestyle factors in relation to obesity phenotype. (22) In accordance with our findings, a cross-sectional study by Naja et al. investigated 350 Lebanese adults and found a positive association between a healthier lifestyle pattern and MHO. This healthier lifestyle pattern was characterized by increased levels of PA (22). However, findings from studies examining the relationship between PA and obesity phenotype are inconsistent. While Farabi et al. and Lopez-Garcia et al. on 99 and 11,520 adults from America and Spain found that the MHO group had higher levels of PA than the MUO group (50, 51). A study on 3,807 adults from Irland did not find any difference in the levels of PA between MUO and MHO groups (52).

The positive association between higher LRS or unhealthier lifestyle and MUO might be due to dietary intake. Participants with lower LRS had a higher intake of whole grains, legumes and vegetables. Vegetables, whole grains and legumes have a high content of fibers, vitamin C, vitamin A, carotenoids, and phytochemicals, which are protective compounds against inflammation and oxidative stress (53). Consistent with our findings, Naja et al.2020 showed that a healthier lifestyle pattern characterized by a higher intake of fruits, vegetables, and legumes was positively linked to MHO (22). Furthermore, a study on 203 Iranian adolescents with overweight and obesity found that higher vegetable intake was negatively associated with MUO risk (54). Also a study on 137 adults with obesity from Brazil found that participants with MHO had a higher diet quality index (55).

A direct link between higher LRS and MUO may be explained by the sociodemgraphic characteristics of study participants. The majority of women with higher LRS had lower levels of education. There is limited evidence on the association between education levels and MHO and MUO. In line with our findings, two studies on 20,430 US adults and 11,520 Spanish adults found that participants with obesity and MUO had lower levels of education (17, 51). A study on 1,073 students from a Mexican university reported that higher education levels were associated with better metabolic health (56). There is abundant evidence indicating that a higher level of education is associated with improved health results and people with higher levels of education are more likely to follow the healthier behavior (56–59). Furthermore, the majority of participants with higher LRS had poor sleep quality. A study on 1,777 US adults reported that participants with sleep complications were less likely to be MHO. While the relationship between sleep and obesity phenotype is still unclear, the evidence shows that chronic lack of sleep results in weight gain through endocrine pathways including leptin and ghrelin which regulate appetite. Poor sleep quality is also associated with higher inflammation, increased sympathetic nervous system activity, insulin resistance, cardiometabolic dysfunction and obesity (60–62).

This study has several limitations that need to be taken into consideration. Firstly, the study design is cross-sectional which is susceptible to reverse causality when examining associations between the LRS and obesity phenotypes. Secondly, dietary intake was assessed through the FFQ, which introduces the potential for memory bias. Thirdly, it is important to note that the study participants were exclusively women from Tehran, making the sample non-representative of the entire Iranian population. Fourthly, this study used the Karelis criteria which does not account for blood pressure. This limitation should be taken into account while comparing the findings of this study with the studies that considered blood pressure to define obesity phenotype. Fifthly, this study used *a priori* approach to create LRS which has limitations. The indices are created based on the current knowledge and the evidence. As a result, this approach is limited by the existing evidence (20). Lastly, the majority of participants were MUO which could influence the findings of this study. This study has a number of strengths. To the best of our knowledge, for the first time, this study applied an *a priori* approach, created the LRS and examined associations between the LRS and obesity phenotypes. The analysis of this study was controlled for several confounding factors. Given the obesity is more prevalent in the women from the EMR than men, this study included Iranian women with overweight and obesity.

## Conclusion

This study, used a novel *a priori* approach through the creation of LRS, showed significant associations between an unhealthier lifestyle, as indicated by a higher LRS, and an increased likelihood of MUO in Iranian women with overweight and obesity. The study highlights the intricate interplay between lifestyle elements and obesity phenotypes, emphasizing the need for comprehensive interventions addressing multiple factors to promote metabolic health. However, the study’s cross-sectional nature introduces limitations, and further research, ideally incorporating longitudinal designs and diverse populations, is warranted to validate these associations and enhance our understanding of the complex relationship between lifestyle and obesity.

## Data Availability

The raw data supporting the conclusions of this article will be made available by the authors, without undue reservation.
