# Supplementary material for: The association between the lifestyle risk score and metabolically healthy and unhealthy obesity phenotype in Iranian women with overweight and obesity: a cross-sectional study
Source: Front Public Health. 2025 Feb 14;13:1490937. doi: 10.3389/fpubh.2025.1490937 (PMC11868931; doi:10.3389/fpubh.2025.1490937)
Supplement: Supplementary file 1 [file Table_1.docx]

**Supplementary Table 1**. The American Heart Association components and standards for scoring

| **Components** | **Criteria for scoring** | **Score range** |
| --- | --- | --- |
| Fruits and vegetables | 0 to ≥4.5 cups/d | 0–10 |
| Fish and shellfish | 0 to ≥7 oz/wk | 0–10 |
| Sodium | ≤1500 to >4500 mg/d | 10–0 |
| sugar-sweetened beverages | ≤36 to >210 fl oz/wk | 10–0 |
| Whole grains | 0 to ≥3 oz/d | 0–10 |
| Nuts, seeds, and legumes | 0 to ≥4 servings/d | 0–10 |
| Processed meats | ≤3.5 to >17.5 oz/wk | 10–0 |
| Saturated fat | ≤7 to >15 (% energy) | 10–0 |
