# Supplementary material for: The association between the lifestyle risk score and metabolically healthy and unhealthy obesity phenotype in Iranian women with overweight and obesity: a cross-sectional study
Source: Front Public Health. 2025 Feb 14;13:1490937. doi: 10.3389/fpubh.2025.1490937 (PMC11868931; doi:10.3389/fpubh.2025.1490937)
Supplement: Supplementary file 2 [file Table_2.docx]

**Supplementary Table 2**. The lifestyle risk score components and standards for scoring

| **LRS components** | **Criteria for scoring** | | | **score** |
| --- | --- | --- | --- | --- |
| Dietary behavior |  | | |  |
|  | AHA diet score ≥ 40 | | | 0 |
|  | AHA diet score < 40 | | | 1 |
| PA )MET-h/week( |  | | |  |
|  | moderate to high PA (> 20 MET-h/week) | | | 0 |
|  | low PA (≤ 20 MET-h/week) | | | 1 |
| Sleep quality |  | | |  |
|  | PSQI ≤ 5 | | | 0 |
|  | PSQI > 5 | | | 1 |
| WHtR |  | | |  |
|  | Non-obesity ≤0.5 | | | 0 |
|  | Obesity >0.5 | | | 1 |
| SES |  | | |  |
| Education* | High educational levels | 1 | High SES  (score ≥2) | 0 |
|  | Low educational levels | 0 |  |  |
| Occupation | Employed | 1 |  |  |
|  | Unemployed | 0 | Low SES  (score < 2) | 1 |
| Income** | High income | 1 |  |  |
|  | Low income | 0 |  |  |
| AHA: American heart association; LRS: Lifestyle risk score; PA: Physical Activity; PSQI: Pittsburgh sleep quality index; SES: Socioeconomic status; WHtR: Waist to hip ratio  *: Participants were categorized into high education level (bachelor degree and higher), low education level (diploma and lower).  **: High income was considered as above poverty line income; low income was considered as below poverty line income (eleven million and five hundred thousand rials for each person was considered as poverty line in 2018). | | | | |
